# TAL effectors mediate high-efficiency transposition of the *piggyBac* transposon in silkworm *Bombyx mori* L

**DOI:** 10.1038/srep17172

**Published:** 2015-11-26

**Authors:** Lupeng Ye, Zhengying You, Qiujie Qian, Yuyu Zhang, Jiaqian Che, Jia Song, Boxiong Zhong

**Affiliations:** 1 College of Animal Sciences, Zhejiang University, Hangzhou 310058, P. R. China

## Abstract

The *piggyBac* (*PB*) transposon is one of the most useful transposable elements, and has been successfully used for genetic manipulation in more than a dozen species. However, the efficiency of *PB*-mediated transposition is still insufficient for many purposes. Here, we present a strategy to enhance transposition efficiency using a fusion of transcription activator-like effector (TALE) and the *PB* transposase (*PBase*). The results demonstrate that the TALE-*PBase* fusion protein which is engineered in this study can produce a significantly improved stable transposition efficiency of up to 63.9%, which is at least 7 times higher than the current transposition efficiency in silkworm. Moreover, the average number of transgene-positive individuals increased up to 5.7-fold, with each positive brood containing an average of 18.1 transgenic silkworms. Finally, we demonstrate that TALE-*PBase* fusion*-*mediated *PB* transposition presents a new insertional preference compared with original insertional preference. This method shows a great potential and value for insertional therapy of many genetic diseases. In conclusion, this new and powerful transposition technology will efficiently promote genetic manipulation studies in both invertebrates and vertebrates.

Transcription activator-like effectors (TALEs) are naturally conserved bacterial effector proteins derived from the *Xanthomonas* genus of plant pathogenic bacteria^1^. To date, TALE proteins have been described as having a simple modular DNA recognition code^2^,^3^, that is composed of repeat domains of 33–35 amino acids. The specificity of TALE is determined by the repeat-variable di-residues (RVDs) at positions 12 and 13 of these repeats^4^,^5^. In recent years, TALE nucleases (TALENs) have been successfully and widely used for the targeted editing of endogenous genes in various species, including yeast^6^, nematodes^7^, frogs^8^, insects^9^,^10^, fish^11^,^12^,^13^,^14^, plants^15^,^16^ and mammals^17^,^18^. TALE proteins have also been engineered with transcriptional regulatory domains to generate artificial transcription factors that can regulate the expression of targeted endogenous genes^16^,^19^,^20^,^21^,^22^,^23^,^24^,^25^. Recent, studies have demonstrated that TALEs can be efficiently exploited to modify epigenomes in a targeted manner^26^,^27^,^28^.

The *piggyBac* (*PB*) transposon, which was originally isolated from the genome of the cabbage looper moth *Trichoplusia ni*^29^, is a type of non-viral vector characterized by a large cargo size^30^, low toxicity^31^ and long-term expression^32^,^33^. *PB* transposon-mediated gene transfer has been successfully performed in various organisms, both invertebrates and vertebrates. Studies in silkworm have benefited from this technology because the silk-gland bioreactor shows great potential for the production of vast quantities of valuable exogenous protein via *PB*-mediated transgenesis. The *PB* transposon system is undoubtedly a powerful genetic manipulation tool for transgenesis and insertional mutagenesis and is currently being applied to the development of a new generation vector for research in human gene therapy and induced pluripotent stem cells^34^,^35^,^36^. However, the efficiency of *PB*-mediated transposition remains limited and unstable. The earliest and most appropriate method for evaluating transposition efficiency in silkworm is to calculate the percentage of G1 positive broods among all G0 moths^37^. Using this method, we have collected and analyzed most of the published transgenic data. However, as the current average for transposition efficiency in silkworm is 8.8% (Supplementary Table S1). The present transposition level must be improved to satisfy the requirements of research and to further promote the application of the *PB* system.

This study presents a monomeric fusion protein engineered from TALE repeat arrays and *PB* transposase (*PBase*) to further exploit the potential functions of TALE. We find that the TALE-*PBase* fusion protein can significantly improve the transposition efficiency of the *PB* system.

## Results

### Transposition efficiency of *piggyBac* in silkworm

**Figure 1:**
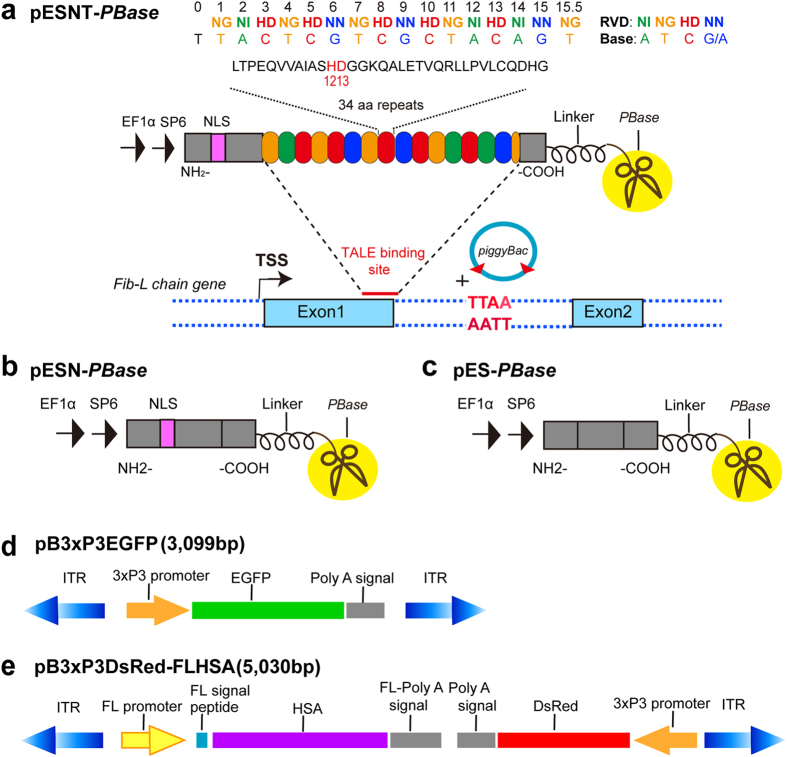
Design and construction of artificial TALE and *PB* transposon plasmids for producing transgenic silkworms. (**a**) Schematic representation of the TALE tandem repeat domain and each repeat monomer, including the two repeat variable di-residues (RVD) at positions 12 and 13 of each amino acid sequence, which determine the base recognition specificity. TALE arrays comprising 16 repeats (colored ovals) fused to the *PB* transposase (*PBase*). EF1α, elongation factor-1 alpha promoter; SP6, a prokaryotic promoter used for high-efficiency mRNA transcription *in vitro*; NLS, nuclear localization signal; *Fib-L* gene, *fibroin light-chain* gene of silkworm; TSS, transcriptional start site; TTAA, the target insertion site of the *PB* transposon. (**b**) Schematic representation of the TALE repeats are deleted in the pESN-*PBase* plasmid based on the pESNT-*PBase* plasmid. (**c**) Both the TALE repeat and the NLS are deleted in the pES-*PBase* plasmid. (**d**,**e**) Diagram of the structure of *PB* transposon plasmids. ITR, inverted terminal repeats of the *PB* transposon; 3 × P3 promoter, an artificial promoter specifically driving reporter gene expression in the ocelli of larvae or compound eyes of moths; FL promoter, silkworm *fibroin light-chain* promoter; HSA, human serum albumin.

**Figure 2:**
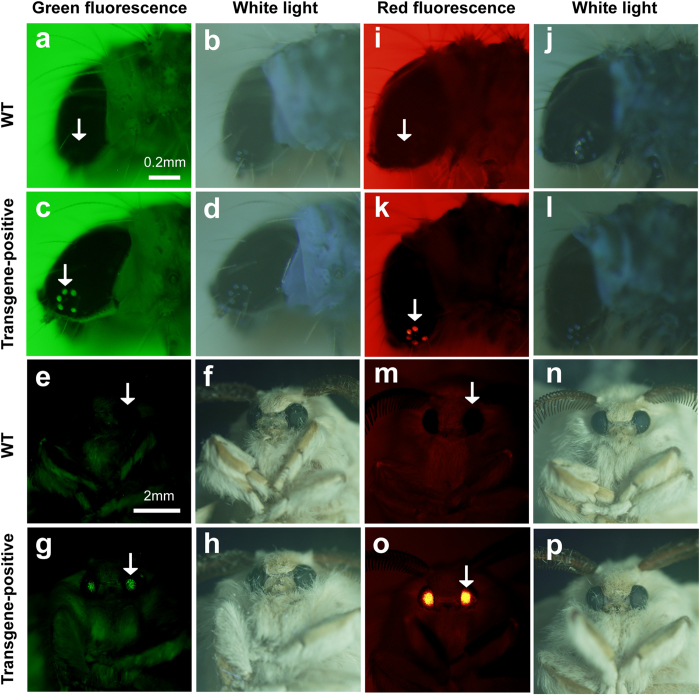
Fluorescence of EGFP or DsRed is specific in the eyes of transgenic silkworm. The first and third columns are viewed under the excitation wavelengths of GFP and DsRed, respectively; the second and fourth columns are white light illumination. The first and third rows are wild-type (WT) larvae on the first day after hatching and moths, respectively; the second and fourth rows are transgenic larvae on the first day after hatching and moths, respectively.

**Figure 3:**
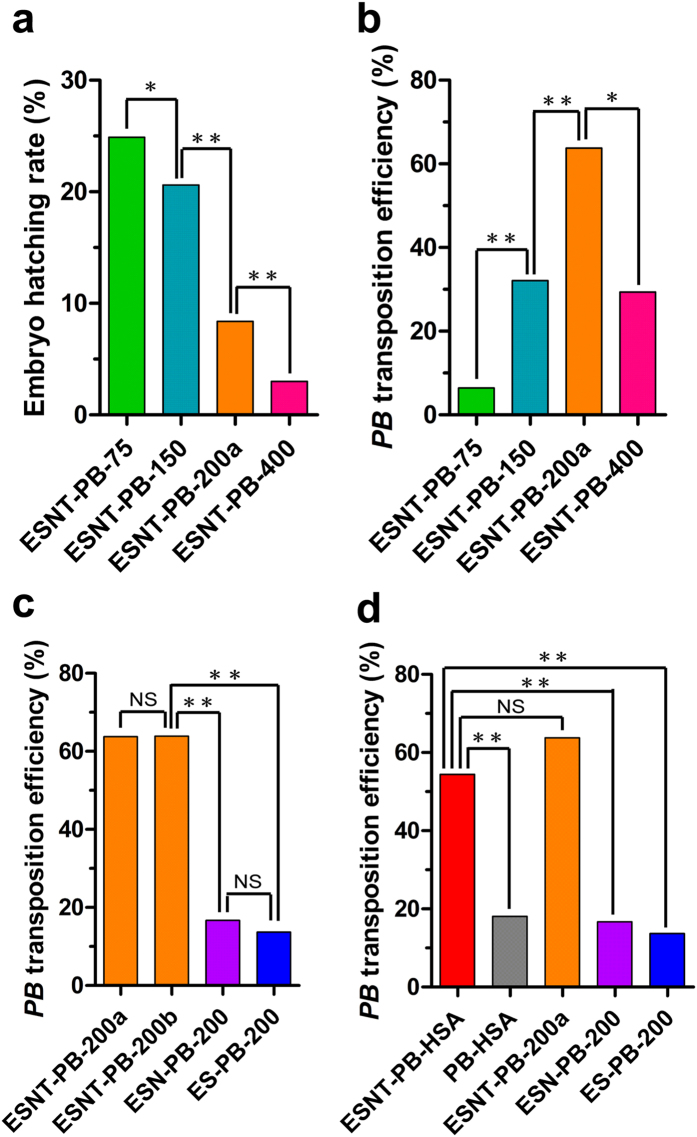
Statistical analysis of the embryos hatching rate and transposition efficiency. (**a**) The hatching rate is directly related to the concentration of pESNT-*PBase* mRNA injected. The hatching rate was significantly reduced by increasing the microinjection concentration. (**b**) Statistical analysis of the transposition efficiency indicates that 200 ng/μL of pESNT-*PBase* mRNA is the optimal concentration for obtaining the highest transposition efficiency and a moderate hatching rate (ESNT-PB-200a series transgenic strains). (**c**,**d**) The TALE-*PBase* fusion can significantly enhance the transposition frequency and maintain transposition at a high level (ESNT-PB-200a, ESNT-PB-200b and ESNT-PB-HSA), even using a larger *PB* transposon plasmid and in a new silkworm strain. *P < 0.05, **P < 0.01, using a significance test for percentage of two samples.

To investigate whether a programmable TALE could improve transposition efficiency, three types of plasmids were constructed: pESNT-*PBase*, consisting of EF1α and SP6 promoters, a nuclear localization signal (NLS), a TALE repeat domain targeting first exon of the *fibroin light-chain* gene and *PBase* (Fig. 1a); pESN-*PBase*, with the TALE sequence deleted (Fig. 1b); and pES-*PBase*, with both NLS and TALE sequence deleted (Fig. 1c). These plasmids were then transcribed *in vitro* to obtain mRNAs and each mRNA was mixed with the pB3 × P3EGFP transposon plasmid (Fig. 1d) and microinjected into fertilized embryos of the *P50* silkworm strain. All of the positive silkworms exhibited a similar phenotype of larvae with green ocelli or moths with green compound eyes (Fig. 2a–h). To identify the optimal microinjection dose, four different concentrations of pESNT-*PBase* mRNA (Table 1) were injected. The hatching rate of the microinjected embryos decreased significantly with increasing concentration of pESNT-*PBase* mRNA (Table 1, Fig. 3a); the highest concentration (400 ng/μL) induced a high embryonic death rate, and only 3.0% of microinjected embryos hatched normally (Table 1). However, neither the highest or lowest mRNA concentration could result in the best transposition efficiency. Overall, injected of 200 ng/μL produced the best transposition efficiency, 63.8% (37/58), which was significantly higher than with the other concentrations (Table 1, Fig. 3b). Using this optimal concentration, we compared the transposition efficiencies of three different plasmids, pESNT-*PBase*, pESN-*PBase* and pES-*PBase*. *PBase* fused to the NLS (pESN-*PBase*) did not significantly improve the transposition efficiency in comparison with *PBase* alone (pES-*PBase*, *P* > 0.05) (Table 1, Fig. 3c). In contrast, the transformation frequency was significantly (*P* < 0.01) improved up to 63.9% (23/36) when pESNT-*PBase*, containing the TALE domain, was injected (Table 1, Fig. 3c). These data demonstrate that the TALE domain robustly improve transposition efficiency.

To demonstrate the universality of pESNT-*PBase*-mediated high-efficiency transposition, we selected a different silkworm strain, *Lan10*, as the transgenic receptor and constructed a larger *PB* transposon as the donor plasmid (Fig. 1e). The reporter gene 3 × P3DsRed was specifically expressed in the eyes of all positive transgenic silkworms (Fig. 2i–p). Indeed, pESNT-*PBase* significantly (*P* < 0.01) improved transposition efficiency, reaching 54.4% (56/103) in ESNT-PB-HSA series transgenic strains compared with PB-HSA strains (18.1%, 21/116) and producing significantly (*P* < 0.01) higher transposition rates than the ESN-PB-200 (16.7%, 8/48) and ES-PB-200 (13.7%, 7/51) transgenic strains (Table 2, Fig. 3d). However, the transposition efficiency of ESNT-PB-HSA series transgenic strains was not significantly different from that of ESNT-PB-200a. These data again confirmed that the TALE-*PBase* fusion could significantly and stably increase transposition frequency, even with a larger cargo size of the transposon plasmid and in a different silkworm strain.

**Figure 4:**
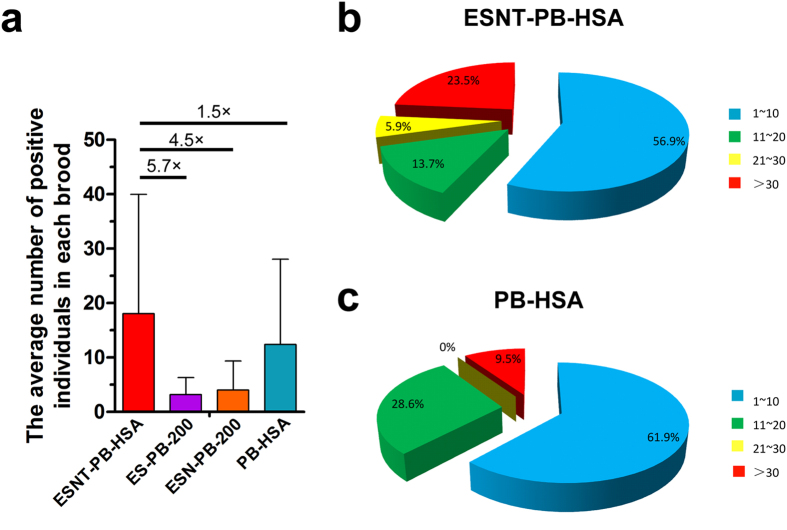
Quantification of transgenic individuals in each brood. (**a**) The average number of positive silkworms in the ESNT-PB-HSA series transgenic strain was 5.7, 4.5 and 1.5 times higher than in ES-PB-200, ESN-PB-200 and PB-HSA, respectively. (**b**,**c**) Total positive broods are divided into four groups to more clearly present the distribution of the number of transgenic individuals in each brood. The pie charts show that the proportions of broods with 21–30 and > 30 transgenic individuals were dramatically improved in the ESNT-PB-HSA series transgenic silkworms compared with PB-HSA.

Furthermore, we compared the numbers of transgenic-silkworms in positive broods among ESNT-PB-HSA, PB-HSA, ESN-PB-200 and ES-PB-200. The average number of positive individuals in each ESNT-PB-HSA series transgenic brood reached 18.1, which was 1.5–5.7 times higher than for the three controls (Fig. 4a). We further performed a more detailed statistical analysis of the number of transgene-positive individuals between ESNT-PB-HSA and PB-HSA. The proportion of broods with more than 20 positive individuals, and especially with more than 30, was dramatically improved in ESNT-PB-HSA (Fig. 4b,c), for which nearly a quarter of positive broods were identified as containing over 30 transgene-positive silkworms. One positive brood (the ESNT-PB-HSA49 transgenic strain) contained 92 transgenic individuals (Supplementary Table S2). In general, TALE-mediated high-efficiency transposition is reflected in both the number of positive broods and the number of transgenic individuals per positive brood.

### Analysis of insertion sites

**Figure 5:**
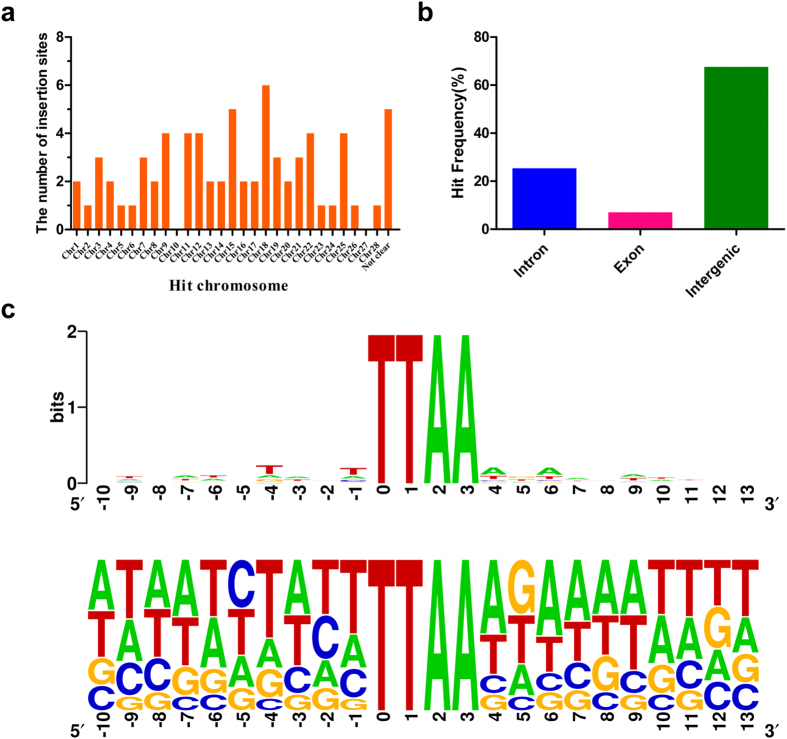
*piggyBac* insertion site analysis in silkworm. (**a**) Distribution of *PB* integration sites on chromosomes. *PB* broadly targeted all chromosomes, except chromosomes10 and 27. (**b**) Analysis of integration sites in genes showing that most *PB* insertions were found in intergenic regions and introns, with only 7.0% appearing in exons. (**c**) Sequence logo analysis of the nucleotide composition of 20 bp flanking sequences around the insertion site “TTAA” based on 71 *PB* insertion events. All of the integrations show TTAA target site specificity, and an enrichment of As and Ts in the flanking sequences is observed. However, the nearest five nucleotides upstream and downstream of the integration sites are changed, presenting a novel pattern of nucleotide composition compared with previous reports.

Previous studies have demonstrated that native *PBase*-mediated gene transposition primarily occurs at TTAA sites and has an insertional preference for AT-rich regions with 5 Ts before and 5 As after the TTAA sites in both insect and mammal^38^,^39^. Our analysis of integration sites indicated that all insertion events occurred in TTAA sites, which were widely distributed among the chromosomes (Fig. 5a, Supplementary Table S3). Most of the transposition events occurred in introns and intergenic regions, with only 7.0% occurring in exons (Fig. 5b). Moreover, a sequence logo analysis indicated that the majority of insertion sites occurred in AT-rich regions (Fig. 5c). However, it is noteworthy that the proximal ten bases around the TTAA site presented a new pattern: the proportions of C, A and G bases at position −5, −3 and+5, respectively, were significantly enriched in comparison with previous studies (Fig. 5c). We believe that the insertional preference of *PB* was substantially altered by using the TALE-*PBase* fusion protein. In theory, the TALE-*PBase* fusion could achieve site-specific integration; however, no insertion events have been identified as occurring in a targeted manner. In the present study, two transposition events, ESNT-PB-200a26 and ESNT-PB-200b18, were identified integrating in the target chromosome (chromosome 14) and scaffold (scaffold 81) (Supplementary Table S3), but which were 278,440 bp and 156,461 bp away from the target integration site, respectively.

## Discussion

The extensive utilization of the *PB* transgenic system has been proven its value in genetic manipulation studies. In recent years, TALEs have demonstrated powerful functions in targeted gene editing, gene regulation and locus-specific histone modifications. So far, no reports have been found from available literatures about the TALE-*PBase* fusions can improve transposition efficiency in other species. The purpose of the present study was to engineer a TALE*-PBase* fusion to improve transposition efficiency. Our results show that *PBase* fused to an NLS cannot significantly enhance transposition efficiency, suggesting that *PBase* may already contain a functional nuclear targeting signal^40^. Therefore, TALE was the most important factor that improved *PB* transposition efficiency, which was enhanced by almost 64%, at least 7 times higher than the current average transposition efficiency in silkworm. In addition, the number of positive individuals in each transgenic brood was maximally increased by up to 5.7-fold. The improvements in these two characteristics present a breakthrough in the optimization of the *PB* transposon system. Moreover, modestly increasing the *PB* cargo size did not produce a significant reduction (*P* > 0.05) in the transposition efficiency. Thus, a *PB* element can simultaneously carry multiple genes to satisfy complex transgenic studies without reducing the frequency of transposition. The *PB*-mediated transgenic efficiency is affected by many factors, so it is hard to get a generally stable transgenic efficiency in the previous studies. In general, the transgenic efficiency of *PB* is insufficient in silkworms. From Supplementary Table S1 we could find that only one study achieved high-efficiency transgenesis (57.61%), but such a result was unstable, which merely appeared once from the four independent transgenic experiments^41^. In our study, the sufficient data demonstrate that the high transgenic efficiency is more stable and repeatable instead of appearing as an accidental phenomenon. So, our results fully illustrate the reliability of the TALE mediates high-efficiency transposition. The native *PBase* may be replaced by this new-type and high-efficient TALE-*PBase* fusion in the future. The next step of the research is to construct more TALE-*PBase* fusion proteins with different targets, which may help us to find more efficient TALE-*PBase* fusions.

This high TALE-mediated transposition frequency may be induced by multiple factors. The fusion of TALE and *PBase* may increase the three-dimensional structural stability of *PBase*, possibly prolonging the period of enzyme activity. As a result, more *PB* transposons are efficiently inserted into the genome. Furthermore, although the gene regulation is complex, it can be accurately long-range controlled by distant regulatory elements, including enhancers and repressors, to coordinated expression of genes in the third dimension^42^,^43^. TALE-mediated gene transposition may also be achieved in a long-range manner in the third dimension (Supplementary Fig. S1). In our study, a monomer TALE was fused to an intact *PBase*, producing an enzyme that can perform all the steps necessary for transposition. We therefore reason that the TALE-*PBase* fusion will combine with many potential candidate loci that may not perfectly matched the preferred TALE site. When TALE recognizes an appropriate site in the genome, *PBase* will execute transposition at multiple candidate “TTAA” sites (Supplementary Fig. S1) because the TALE-*PBase* fusion protein is larger than the native *PBase* protein and thus can perform transposition at a larger spatial scale. As some candidate “TTAA” sites may be hundreds of kilobases away from the TALE binding site (Supplementary Fig. S1a) or may even be located on different chromosomes (Supplementary Fig. S1b), the three-dimensionality of TALE-mediated transposition contributes to improving transposition efficiency.

Using a *PBase* coupled with a TALE, the first site-specific insertion of *PB* was recently identified in ~0.010-0.014% of stably transfected human cells^44^. Although this demonstrates that targeted insertions can be achieved, but the efficiency is still very low. Here, we have not found site-specific integration events, but TALE-mediated transposition has been shown to slightly alter the original insertion preference of *PB* (Fig. 5c). So, site-specific integration is still a challenge, but it may be markedly improved if the ability of *PBase* to recognize its target locus is weakened without compromising its catalytic function. Our results provide important clues for developing a high-efficiency insertional therapy tool which has shown greatly potential value in genetic disease therapy.

In summary, our study first demonstrates that the TALE-*PBase* fusion powerfully improves transposition efficiency in silkworms. This discovery introduces a new area for the application of TALE in research. To date, the *PB* system and TALE have been widely applied in various species of invertebrates and vertebrates. Thus, we believe that a TALE-*PBase* fusion will also function well in organisms other than silkworms. Our study will greatly promote *PB*-mediated genetic manipulation studies, including the generation of transgenic animals, insertional mutagenesis and gene therapy.

## Methods

### Construction of TALE-*PBase* fusion and transgenic plasmids

Target TALE assembly was performed using a FastTALE^TM^ TALEN kit (SIDANSAI biotechnology CO., LTD) according to the manufacturer’s instructions. The targeted binding site was in the first exon of the *fibroin light chain* (*Fib-L*) gene (chromosome14, scaffold81) (Supplementary Fig. S2), the expression product of which is the main component of fibroin in silkworm. The *PBase* gene was then engineered into the TALE vector, and the constructed plasmid was named pESNT-*PBase* due to the inclusion of EF1α, a ubiquitous promoter which exhibits a strong activity in eukaryotic cells, and SP6 promoters, an NLS, a TALE repeat domain and *PBase*. In addition, two control plasmids, pESN-*PBase* (TALE deleted) and pES-*PBase* (both TALE and NLS deleted), were constructed from pESNT-*PBase*. The mRNA of these vectors was synthesized *in vitro* using an SP6 mMESSAGE mMACHINE Kit (Ambion). The donor plasmids pB3 × P3EGFP and pB3 × P3DsRed-FLHSA (FLHSA, human serum albumin gene driven by a *Fib-L* promoter) were constructed based on pBA3EGFP transposon plasmid. The marker gene (EGFP or DsRed) was controlled by a 3 × P3 promoter, an artificial promoter specifically driving expression in the eyes and nervous tissues, which is useful for the screening of positive individuals. All plasmids were extracted using the Quick Plasmids Miniprep kit (Invitrogen), followed by further purification to remove residual RNase A, as described in the SP6 mMESSAGE mMACHINE Kit (Ambion). Briefly, plasmid DNA was treated with 0.5% SDS and proteinase K (200 μg/mL) for 30 min at 50 °C, followed by phenol/chloroform extraction (using an equal volume) and precipitation with 2 volumes of ethanol. Finally, the samples were centrifuged at 23,500 *g* for 15 min to harvest the purified DNA.

### Transgenesis and screening of silkworms

The experimental animals *P50* (*Dazao*) and *Lan10*, multivoltine silkworm strains with diapause ability, were reared on fresh mulberry leaves under standard conditions (25 °C, 80% R.H). Embryo microinjection and the screening of positive silkworms were performed as described previously^37^,^45^. Briefly, zygotes were collected promptly, and microinjection was completed within 4 h after oviposition. The helper plasmid pESNT-*PBase* mRNA was mixed with the donor plasmid pB3 × P3EGFP based on the actual concentration before injection into one-cell-stage fertilized eggs. The microinjected eggs were cultured under standard conditions, and each surviving moth was mated with wild-type moths to obtain G1 generations. Finally, positive individuals were screened from G1 broods based on the presence of green or red eyes using a fluorescence microscope SZX16 (Olympus). The procedures for the other two helper plasmid mRNAs, pESN-*PBase* and pES-*PBase*, were similar to the protocol described above.

### Statistics of published transposition efficiency

The most appropriate method for the evaluation of silkworm transposition efficiency is to calculating the percentage of G1 positive broods in total G0 moths^37^. However, in some studies, transgenic G0 moths were mated with each other or mated within the same family to generate G1 broods, and the ratio of G1 positive broods/total G1 broods was calculated as the final transposition efficiency. This computation method led to transposition efficiencies that were nearly twice those obtained using the former method. Therefore, we calibrated these transposition efficiencies by halving them in an effort to standardize the method for computing transposition efficiency.

### Analysis of insertion sites

Inverse PCR analysis^37^ was conducted after genomic DNA was isolated from each positive transgenic silkworm strain. Briefly, 1 μg of total genomic DNA was digested with *Sau*3A I at 37 °C for 2 h and then self-ligated overnight at 16 °C using T4 DNA ligase (TAKARA). A 25–50 ng sample of ligated products were amplified using *EX Taq* polymerase (TAKARA) and specific primers (pB3 × P3EGFP left arm primer pair, 5′-ATCAGTGACACTTACCGCATTGACA-3′ and 5′-TGACGAGCTTGTTGGTGAGGATTCT-3′; pB3 × P3EGFP right arm primer pair, 5′-TACGCATGATTATCTTTAACGTA-3′ and 5′-GGGGTCCGTCAAAACAAAACATC-3′). The PCR program was conducted with a 3 min denaturation cycle at 96 °C followed by 40 cycles of 30 s at 96 °C, 30 s at 60 °C, and 2 min at 72 °C, and a final extension at 72 °C for 10 min. The amplified PCR products were sequenced after cloning in pMD19-T (TAKARA) to identify the exact sites of *PB* insertion into silkworm chromosomes. Two pairs of primers were designed for PCR detection of the same insertion sites. The forward primer 5′-CCTGTGGTAGATTCTGCGAAG-3′ and the reverse primer 5′-CCTTTACATGAGCCTGACGTCA-3′ were used for identification of ESNT-PB-200a1 and ESNT-PB-200a17a transgenic strains; the forward primer 5′-TCTGTCGCAAGTCGCCAGTTT-3′ and reverse primer 5′-CCTTTACATGAGCCTGACGTCA-3′ were used for the identification of ESNT-PB-200a31 and ESNT-PB-200b7 transgenic strains.

## Additional Information

**How to cite this article**: Ye, L. *et al.* TAL effectors mediate high-efficiency transposition of the *piggyBac* transposon in silkworm *Bombyx mori* L. *Sci. Rep.* **5**, 17172; doi: 10.1038/srep17172 (2015).

## Supplementary Material

Supplementary Information

## Figures and Tables

**Table 1:** TALE-mediated *piggyBac* transposition efficiency in *P50* strain.

Transgenic strain	Injected mRNA/concentration (ng/μL)	Microinjected embryos	Hatched embryos (%)	G1 generation broods	Examined G1 broods	EGFP-positive G1 broods (From examined broods)	Percentage of transposition efficiency (%)
ESNT-PB-75	pESNT-*PBase*/75	770	192 (24.9)	89	47	3	6.4
ESNT-PB-150	pESNT-*PBase*/150	1100	227 (20.6)	83	56	18	32.1
ESNT-PB-200a	pESNT-*PBase*/200	850	71 (8.4)	58	58	37	63.8
ESNT-PB-400	pESNT-*PBase*/400	1450	44 (3.0)	17	17	5	29.4
ESNT-PB-200b	pESNT-*PBase*/200	900	75 (8.3)	36	36	23	63.9
ESN-PB-200	pESN-*PBase*/200	900	203 (22.6)	110	48	8	16.7
ES-PB-200	pES-*PBase*/200	800	93 (11.6)	119	51	7	13.7

The microinjected *PB* transposon plasmid was pB3 × P3EGFP and the concentration was 300 ng/μL.

**Table 2:** TALE-mediated *piggyBac* transposition efficiency in *Lan10* strain.

Transgenic strain	Injected mRNA or DNA/concentration (ng/μL)	Microinjected embryos	Hatched embryos (%)	G1 generation examined broods	DsRed -positive G1 broods	Percentage of transposition efficiency (%)
ESNT-PB-HSA	pESNT-*PBase*/200	1100	234 (21.3)	103	56	54.4
PB-HSA	*PBase* (DNA)/200	1100	258 (23.4)	116	21	18.1

The microinjected *PB* transposon plasmid was pB3 × P3DsRed-FLHSA and the concentration was 200 ng/μL.
