# Supplementary material for: TAL effectors mediate high-efficiency transposition of the piggyBac transposon in silkworm Bombyx mori L
Source: Sci Rep. 2015 Nov 26;5:17172. doi: 10.1038/srep17172 (PMC4660427; doi:10.1038/srep17172)
Supplement: Supplementary Information [file srep17172-s1.pdf]

TAL effectors mediate high-efficiency transposition of the *piggyBac* transposon in silkworm *Bombyx mori* L.

Lupeng Ye[1], Zhengying You[1], Qiujie Qian[1], Yuyu Zhang[1], Jiaqian Che[1], Jia Song[1] & Boxiong Zhong[1]*

[1] College of Animal Sciences, Zhejiang University, Hangzhou 310058, P. R. China

*Corresponding author. Tel:/Fax: +86-571-86971302; E-mail: bxzhong@zju.edu.cn

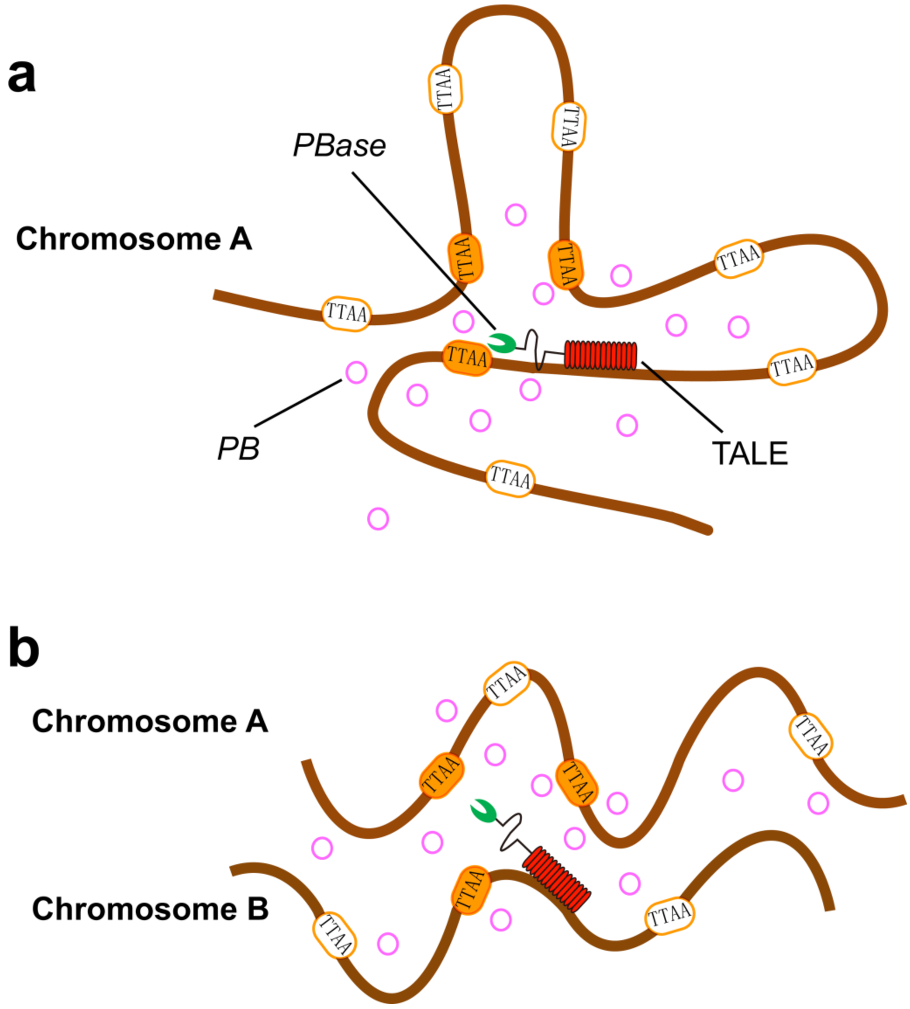

Supplementary Figure S1. Three-dimensional TALE-mediated transposition. (**a**) The occurrence of *PB* transposition within the same chromosome. (**b**) The occurrence of *PB* transposition between two different chromosomes. There are many “TTAAs” distributed throughout the genome, and the orange ellipses represent candidate “TTAAs” for transposition. TALE, transcription activator-like effector; *PB*, *piggyBac* transposon; *PBase*, *piggyBac* transposase.

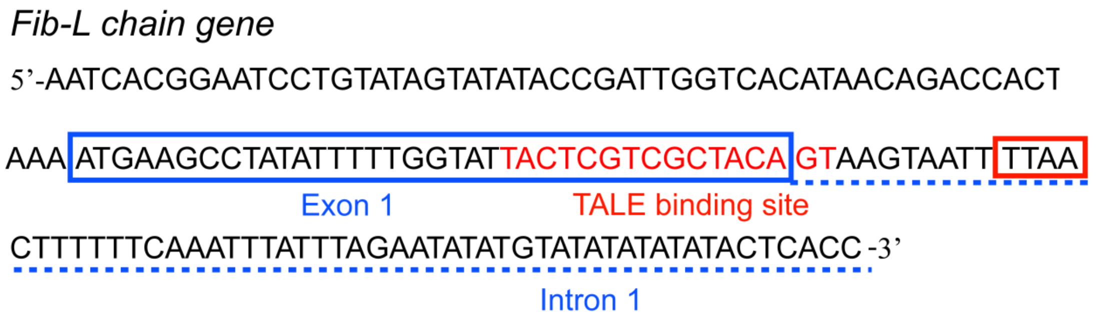

Supplementary Figure S2. The target binding site of TALE. The target binding site of TALE is located on the first exon of the *Fibroin light chain* (*Fib-L*) gene (in the blue frame). The sequence in red is the recognition site of the engineered TALE; “TTAA” in the red frame is the candidate integration site of the *PB* transposon. The sequence above the blue dashed line is the first intron of the *Fib-L* gene (partial sequence).

Supplementary Figure S3. Amino acid sequence of the TALE-PBase fusion protein

MAPKKKRKVGIHGVPMVDLRTLGYSQQQQEKIKPKGGRQSRSITKRWWVM
GLHMPTSSLVAAPCSPWHGRRQVPGHDSALPEATHEAIVGVGKQWSGARALE
ALLTVAGELRGPPLQLDTGQLLKIAKRGGVTAVEAVHAWRNALTGAPLNLTPE
QVVAIASNGGGKQALETVQRLLPVLCQAHGLTPEQVVAIASNIGGKQALETV
QRLLPVLCQAHGLTPEQVVAIASHDGGKQALETVQRLLPVLCQAHGLTPEQV

VAIASNGGGKQALETVQRLLPVLCQAHGLTPEQVVAIASHDGGKQALETVQR
LLPVLCQAHGLTPEQVVAIASNNGGKQALETVQRLLPVLCQAHGLTPEQVVAI
ASNGGGKQAFGNRTTMLPVLCQAHGLTPEQVVAIASHDGGKQALETVQRLLP
VLCQAHGLTPEQVVAIASNNGGKQALETVQRLLPVLCQAHGLTPEQVVAIAS
HDGGKQALETVQRLLPVLCQAHGLTPEQVVAIASNGGGKQALETVQRLLPVL
CQAHGLTPEQVVAIASNIGGKQALETVQRLLPVLCQAHGLTPEQVVAIASHDG
GKQALETVQRLLPVLCQAHGLTPEQVVAIASNIGGKQALETVQRLLPVLCQA
HGLTPEQVVAIASNNGGKQALETVQRLLPVLCQAHGLTPEQVVAIASNGGGRP
ALESIVAQLSRPDPALAALTNDHLVRWPASADDPPWMRLKKGLPHAPALIKRT
NRRIPERTSHRVAGSMGCSLDDEHILSALLQSDDELVGEDSDSEISDHVSEDDV
QSDTEEAFIDEVHEVQPTSSGSEILDEQNVIEQPGSSLASNRILTLPQRTIRGKN
KHCWSTSKSTRRSRVSALNIVRSQRGPTRMCRNIYDPLLCFKLFFTDEIISEIVK
WTNAEISLKRRESMTGATFRDTNEDEIYAFFGILVMTAVRKDNHMSTDDLFDR
SLSMVYVSVMSRDRFDFLIRCLRMDDKSIRPTLRENDVFTPVRKIWDLFIHQCI
QNYTPGAHLTIDEQLLGFRGRCPFRMYIPNKPSKYGIKILMMCDSGTKYMING
MPYLGRGTQTNGVPLGEYYVKELSKPVHGSCRNITCDNWFTSIPLAKNLLQE
PYKLTIVGTVRSNKREIPEVLKNSRSRPVGTSMFCFDGPLTLVSYKPKPAKMV
YLLSSCDEDASINESTGKPQMVMYYNQTKGGVDTLDQMCSVMTCSRKTNR
WPMALLYGMINIACINSFIIYSHNVSSKGEKVQSRKKFMRNLYMSLTSSFMRK
RLEAPTLKRYLRDNISNILPNEVPGTSDDSTEEPVMKKRTYCTYCPSKIRRKAN
ASCKKCKKVICREHNIDMCQSCF*

SV40 NLS
TALE
PBase

Supplementary Table S1. Statistical analysis of silkworm transposition efficiency in previous studies

References (see last page)	Authors and publish time	Mating methods described in the papers	Transposition efficiency in the papers (%)	Transposition efficiency after calibration (%)
1	Tamura et al., 2000	G0 moths are sibling mated or backcrossed	0.7 (3/424)	0.7
			3.9 (9/230)	3.9

2	Tomita et al., 2003	G0 moths are intercrossed	25.8 (42/163)	12.9[a]
			18.3 (24/131)	9.15[a]
			27.6 (35/127)	13.8[a]
3	Imamura et al., 2003	G0 moths are sibling mated	2.7 (3/112)	1.35[a]
			2.5 (3/121)	1.25[a]
			11.2 (19/169)	5.06[a]
4	Adachi et al., 2006	G0 moths are mated within the same family	18.3 (46/251)	9.1[a]
5	Hino et al., 2006	No description	13.4	-
6	Ogawa et al., 2007	G0 moths are mated within the same family	7.88 (28/355)	3.99[a]
7	Zhong et al., 2007	G0 moths are backcrossed	14.2 (46/324)	14.2
			2.62 (7/267)	2.62
			1.72 (2/116)	1.72
8	Wen et al., 2010	G0 moths are mated randomly	8.07 (13/161)	4.03[a]
			0 (0/112)	0[a]
9	Zhao et al., 2010	G0 moths are mated with each other or backcrossed	1.47 (1/68)	1.47
			0.25 (1/400)	0.25
			6.58 (5/76)	6.58
10	Zhuang et	G0 moths are	4.9 (18/367)	4.9

	al., 2010	backcrossed	0 (0/32)	0
			18.5 (5/27)	18.5
11	Sato et al., 2012	G0 moths are mated with each other	4.3 (7/162)	2.15[a]
			23.4 (11/47)	11.7[a]
12	Jiang et al., 2012	G0 moths are mated with each other or backcrossed	16.67 (1/6)	-
			20.00 (1/5)	-
			61.54 (8/13)	-
			50.00 (1/2)	-
13	Jiang et al., 2013	G0 moths are sibling mated	17.20 (5/29)	8.60[a]
			5.88 (1/17)	2.94[a]
			7.69 (1/13)	3.85[a]
			7.17 (1/14)	3.59[a]
			12.50 (1/8)	6.25[a]
			8.33 (1/12)	4.17[a]
14	Jiang et al., 2014	G0 moths are mated with each other or backcrossed to nontransgenic moths	29.77 (39/131)	-
			18.49 (27/146)	-
			27.50 (22/80)	-
			13.56 (8/59)	-
			38.67 (29/75)	-
			11.63 (5/43)	-
			9.68 (3/31)	-

			5.41 (2/37)	-
15	Duan et al., 2013	G0 moths are backcrossed	5.3 (1/19)	5.3
16	Wang et al., 2013	G0 moths are sibling mated	42.9 (9/21)	21.4[a]
			40.0 (8/20)	20.0[a]
			13.0 (3/23)	6.5[a]
17	Tan et al., 2013	G0 moths are backcrossed	12.6 (12/95)	12.6
18	Ma et al., 2013	Female G0 moths are allowed to mate with uninjected male moths	57.61 (53/92)	57.61
			21.21 (14/66)	21.21
			2.00 (1/50)	2.00
			32.28 (92/285)	32.28
19	Deng et al., 2013	G0 moths are mated with each other	4.0 (5/126)	2.0[a]
20	Tsubota et al., 2014	No description	8.9 (5/56)	-
			21.7 (13/60)	-
			11.4 (4/35)	-
21	Xu et al., 2014	G0 moths are mated with each other	25 (2/8)	12.5[a]
22	Xu et al., 2014	G0 moths are mated with each other	14.5 (16/110)	7.25[a]

				Mean: 8.8

Footnotes: [a] Transgenic G0 moths were mated with each other to generate G1 broods; the total G0 transgenic moths should include both male and female G0 moths, but in these studies, only G1 broods were counted (each G1 brood comes from a pair of male and female G0 moths) as the number of total G0 transgenic moths. Thus, the transposition efficiency is calibrated by halving the value.

"-", inappropriate for statistical analysis, because these studies did not clearly indicate how many G0 moths were mated with each other or backcrossed to wild-type moths.

Supplementary Table S2. The number of positive silkworms in each brood

Transgenic silkworm strains	Positive individual number in broods	Transgenic silkworm strains	Positive individual number in broods
ESNT-PB-HSA1	33	PB-HSA1	4
ESNT-PB-HSA2	26	PB-HSA2	4
ESNT-PB-HSA3	31	PB-HSA3	8
ESNT-PB-HSA4	1	PB-HSA4	15
ESNT-PB-HSA5	2	PB-HSA5	2
ESNT-PB-HSA6	73	PB-HSA6	20
ESNT-PB-HSA7	10	PB-HSA7	13
ESNT-PB-HSA8	15	PB-HSA8	7
ESNT-PB-HSA9	4	PB-HSA9	15
ESNT-PB-HSA10	33	PB-HSA10	2
ESNT-PB-HSA11	8	PB-HSA11	2
ESNT-PB-HSA12	2	PB-HSA12	20
ESNT-PB-HSA13	6	PB-HSA13	11
ESNT-PB-HSA14	11	PB-HSA14	9
ESNT-PB-HSA15	14	PB-HSA15	2
ESNT-PB-HSA16	7	PB-HSA16	1
ESNT-PB-HSA17	53	PB-HSA17	72
ESNT-PB-HSA18	34	PB-HSA18	7

ESNT-PB-HSA19	2	PB-HSA19	3
ESNT-PB-HSA20	8	PB-HSA20	2
ESNT-PB-HSA21	6	PB-HSA21	35
ESNT-PB-HSA22	1		
ESNT-PB-HSA23	1		
ESNT-PB-HSA24	41		
ESNT-PB-HSA25	5		
ESNT-PB-HSA26	8		
ESNT-PB-HSA27	2		
ESNT-PB-HSA28	75		
ESNT-PB-HSA29	3		
ESNT-PB-HSA30	2		
ESNT-PB-HSA31	9		
ESNT-PB-HSA32	3		
ESNT-PB-HSA33	1		
ESNT-PB-HSA34	20		
ESNT-PB-HSA35	5		
ESNT-PB-HSA36	1		
ESNT-PB-HSA37	75		
ESNT-PB-HSA38	28		
ESNT-PB-HSA39	12		

ESNT-PB-HSA40	4	
ESNT-PB-HSA41	32	
ESNT-PB-HSA42	18	
ESNT-PB-HSA43	2	
ESNT-PB-HSA44	23	
ESNT-PB-HSA45	5	
ESNT-PB-HSA46	10	
ESNT-PB-HSA47	11	
ESNT-PB-HSA48	45	
ESNT-PB-HSA49	92	
ESNT-PB-HSA50	4	
ESNT-PB-HSA51	4	
Average number of positive individuals in broods	18.1	12.1

Supplementary Table S3. Insertion sites of ESNT-PB-200a and ESNT-PB-200b series transgenic strains

Transgenic strains	Hit chromosome position	Hit scaffold position	Insertion position	5'-flanking genomic DNA sequence	Vector	3'-flanking genomic DNA sequence
ESNT-PB-200a1	Chr25	144	Intergenic	ATTCGATGTACCTTAA	*piggyBac*	TTAAACTTTTATGTATT
ESNT-PB-200a3	Chr19	36	Intron	AGACAATTTACATTAA	*piggyBac*	TTAACATTCTTTTTAA
ESNT-PB-200a4	Chr18	2	Intergenic	AGTTTTTTATACTTTAA	*piggyBac*	TTAAATAAATGGCTCT
ESNT-PB-200a5a	Chr3	63	Exon	GCAGGTCCTCAATTAA	*piggyBac*	TTAAAAGCCGTATCG
ESNT-PB-200a5b	Not clear	293	Intergenic	GCTATTTACGAATTTAA	*piggyBac*	TTAAAATAATTATTCG
ESNT-PB-200a6a	Chr7	45	Intergenic	CAGTTTTTGCTTTAA	*piggyBac*	TTAATTTTGTACGTAT
ESNT-PB-200a6b	Chr9	87	Intron	AAATCTGGTTTTTTAA	*piggyBac*	TTAAAGAAGATACG
ESNT-PB-200a7a	Chr22	69	Intergenic	GTGTGAAGTAGTTAA	*piggyBac*	TTAAATAAAAATGTC
ESNT-PB-200a7b	Chr18	2	Intergenic	GTTTTGATGTTATTAA	*piggyBac*	TTAAGGCTGTCCGC

ESNT-PB-200a8	Chr18	2	Intron	TAAACATTGACATTAA	*piggyBac*	TTAATACCTAGTCGA
ESNT-PB-200a9a	Chr11	16	Intron	ACGTCAGCTATGTTAA	*piggyBac*	TTAACGATGGATTTG
ESNT-PB-200a9b	Chr4	5	Intergenic	ACACTTAAGTATCTTAA	*piggyBac*	TTAAGGTAGCGTCA
ESNT-PB-200a10a	Chr11	24	Intergenic	AGCTTATCTATATATTAA	*piggyBac*	TTAATACGTGGCAA
ESNT-PB-200a10b	Chr13	1	Intergenic	ATGCCAAATACCTTAA	*piggyBac*	TTAAATAAAACATAC
ESNT-PB-200a12	Not clear	317	Intron	CACTACTCACCTTTAA	*piggyBac*	TTAAGGATGAAGCCA
	Chr25	89	Intron	GATCTATCTTATCTTAA	*piggyBac*	TTAAATTAGATTGAA
ESNT-PB-200a13	Chr3	17	Intergenic	ACAGTTTTCATATTTAA	*piggyBac*	TTAACTAGATTTTTA
ESNT-PB-200a14	Chr23	31	Exon	TGTTCCAACCGATTAA	*piggyBac*	TTAAATATTCTCTTC
ESNT-PB-200a15a	Chr9	41	Intergenic	TCGCTGAATAATTTTAA	*piggyBac*	TTAATCATCTAAGATA
	Chr11	16	Intergenic	CCATAGTTGCATTTAA	*piggyBac*	TTAAAGACATCTATC
ESNT-PB-200a16	Not clear	165	Intron	ATAAAAACAACATTAA	*piggyBac*	TTAAATTTGTACAGTT

ESNT-PB-200a17a	Chr25	144	Intergenic	ATTCGATGTACCTTAA	*piggyBac*	TTAAACTTTTATGTAT
ESNT-PB-200a17b	Chr12	6	Intron	ACCCCCCGACATTAA	*piggyBac*	TTAATACTCACAATA
	Chr3	17	Intergenic	TAAAAGCATTTTTAA	*piggyBac*	TTAATTATGATTCTTA
	Chr25	89	Intergenic	ATTTGATATTATTTAA	*piggyBac*	TTAAGTAGCAACGCT
ESNT-PB-200a18	Chr7	136	Intron	ATATTGCTTTGCTTAA	*piggyBac*	TTAAGGCTTCGTCAA
ESNT-PB-200a19	Chr16	124	Intron	CACTGTAGCTCTTTAA	*piggyBac*	TTAATTATAAACACAC
ESNT-PB-200a20	Chr21	7	Intergenic	TTTGGCTTCGTAATTAA	*piggyBac*	TTAACGAAGAGTCCT
ESNT-PB-200a21	Chr26	34	Intergenic	TCATTGCACCTATTAA	*piggyBac*	TTAATAGCCCGGTAC
ESNT-PB-200a23	Chr5	54	Exon	GTAAATAGTTTGTTAA	*piggyBac*	TTAAATAAATGTTATT
ESNT-PB-200a24	Chr7	15	Intron	AGTTTTTTTTTTTTAA	*piggyBac*	TTAATGAATAGAGAC
ESNT-PB-200a25	Chr12	6	Intergenic	TAAAGTGTTGTTTAA	*piggyBac*	TTAATGGTCTTAAAAA
ESNT-PB-200a26	Chr14	81	Intergenic	AACAATAATATTTAA	*piggyBac*	TTAACAAAAGCATTTA

ESNT-PB-200a27	Chr22	133	Intron	GTAAAGCTGCTTTAA	*piggyBac*	TTAACGTGTATTCAGA
ESNT-PB-200a28	Chr18	2	Intergenic	AATGCGCTACTTTAA	*piggyBac*	TTAATGACTTATAATAT
ESNT-PB-200a29a	Chr16	4	Intergenic	ACCATGTGTAACTTAA	*piggyBac*	TTAAAAACACTCTTGT
ESNT-PB-200a29b	Chr6	49	Intergenic	ACTCAGGGAGTTTAA	*piggyBac*	TTAATGGAGAAGGTAG
ESNT-PB-200a30	Chr2	27	Intergenic	TTATAATGTGATATTAA	*piggyBac*	TTAATGAATTTTGTTTT
ESNT-PB-200a31	Chr19	60	Intergenic	TTTCCTTATTATTTAA	*piggyBac*	TTAAACTGCGTCAGAC
ESNT-PB-200a32a	Chr28	47	Intergenic	TGGCATTTGCTCTTAA	*piggyBac*	TTAAAGAAAGGCCGT
ESNT-PB-200a32b	Not clear	228	Intergenic	ATATTATTTACTTTTAA	*piggyBac*	TTAAAGACACGGACG
ESNT-PB-200a33	Chr22	18	Intergenic	CTAAACATGGCTTAA	*piggyBac*	TTAAATAAATAAGGGT
ESNT-PB-200a34a	Chr24	43	Intergenic	TAATTCACGTATTTAA	*piggyBac*	TTAAACAAGTTATTTA
ESNT-PB-200a34b	Chr12	119	Intron	GAGACTGTTCTTTTAA	*piggyBac*	TTAAATTCTAAATAAT
ESNT-PB-200a35a	Chr1	26	Intergenic	TAACAATTTTGTTTAA	*piggyBac*	TTAAAGAAATCGGCT

ESNT-PB-200a35b	Chr12	6	Intergenic	CTATTAGGTACTTTAA	*piggyBac*	TTAAATAATCGTTTTC
ESNT-PB-200a36	Chr15	64	Intergenic	CCTCCTGATCCATTAA	*piggyBac*	TTAACGGTGCTTTTA
ESNT-PB-200a37	Chr20	99	Intergenic	TGTTTCGCTTCTGTTAA	*piggyBac*	TTAATGAAAATAACC
ESNT-PB-200b1	Chr13	1	Intergenic	TTCATTATATGTTTAA	*piggyBac*	TTAAATACAAAGACTC
ESNT-PB-200b2	Chr15	42	Intron	ACAGTGCCAATTTTAA	*piggyBac*	TTAAAAAAAATGTATT
ESNT-PB-200b4	Chr20	99	Intergenic	ATTCTGGCAGCTTTAA	*piggyBac*	TTAATAACAATTGTAT
ESNT-PB-200b5	Chr18	2	Intron	GGATCTACACTATTAA	*piggyBac*	TTAAATTAATAGGGTT
ESNT-PB-200b6	Chr9	77	Intron	CCTTTAATATTGCTTAA	*piggyBac*	TTAATTGAATGCGAA
ESNT-PB-200b7	Chr19	60	Intergenic	TTTCCTTATTATTTAA	*piggyBac*	TTAAACTGCGTCAGAC
ESNT-PB-200b8	Chr8	58	Intergenic	ACGTGAACTACTTTAA	*piggyBac*	TTAATTATTCACAGTC
ESNT-PB-200b10	Chr15	3	Intergenic	ATCGTGAAAAGCTTAA	*piggyBac*	TTAAAGCATAAACTA
ESNT-PB-200b11	Chr11	16	Intergenic	ACCGTTAACGCCATTAA	*piggyBac*	TTAACACAGAATCTT

ESNT-PB-200b12	Chr1	142	Intergenic	TTACAAAACCATCTTAA	*piggyBac*	TTAAAGCCAATATGG
ESNT-PB-200b13	Chr8	19	Exon	TTATGATATTTGTTTTAA	*piggyBac*	TTAAGTTTGTAAT
ESNT-PB-200b14	Not clear	192	Intergenic	TGATAAAACTGTATTAA	*piggyBac*	TTAAAATATTTTTGAC
ESNT-PB-200b15	Chr15	3	Intergenic	CTTGATAACTTGTTTAA	*piggyBac*	TTAAAGGATTCTTTAG
ESNT-PB-200b16	Chr18	2	Intron	TATGCATACATACTTAA	*piggyBac*	TTAAGGTCAATCGGG
	Chr21	7	Intergenic	CAACTAACCGCTTTAA	*piggyBac*	TTAAAGACACAACAA
ESNT-PB-200b17	Chr22	61	Intergenic	GTTGTGTTGTTTTTAA	*piggyBac*	TTAAAACGGTTTTTT
ESNT-PB-200b18	Chr14	81	Intergenic	TTAAAACATTTTTTTAA	*piggyBac*	TTAAATAAGTAATTTC
ESNT-PB-200b19	Chr17	114	Intergenic	CAACAATGTCGCTTAA	*piggyBac*	TTAAACTTAATAGCCT
ESNT-PB-200b20	Chr21	181	Intergenic	AATCACTATTATGTTAA	*piggyBac*	TTAATTGGTGTTGGAT
ESNT-PB-200b21	Chr15	64	Intron	CGGTACGTTTGTGTTAA	*piggyBac*	TTAACTAGTTCGCGC
ESNT-PB-200b22	Chr17	154	Exon	ATGCAAAACACTTTAA	*piggyBac*	TTAATGAGTATTGATA

ESNT-PB-200b23a	Chr9	14	Intergenic	TCAAAGGCAACATTAA	*piggyBac*	TTAAGGGAACGTACC
ESNT-PB-200b23b	Chr4	13	Intergenic	CAGTGTTTGTTTTTAA	*piggyBac*	TTAAAAAAAAAAACA

References

1 Tamura, T. *et al.* Germline transformation of the silkworm Bombyx mori L. using a piggyBac transposon-derived vector. *Nat Biotechnol* **18**, 81-84 (2000).

2 Tomita, M. *et al.* Transgenic silkworms produce recombinant human type III procollagen in cocoons. *Nat Biotechnol* **21**, 52-56 (2003).

3 Imamura, M. *et al.* Targeted gene expression using the GAL4/UAS system in the silkworm Bombyx mori. *Genetics* **165**, 1329-1340 (2003).

4 Adachi, T., Tomita, M., Shimizu, K., Ogawa, S. & Yoshizato, K. Generation of hybrid transgenic silkworms that express Bombyx mori prolyl-hydroxylase alpha-subunits and human collagens in posterior silk glands: Production of cocoons that contained collagens with hydroxylated proline residues. *J Biotechnol* **126**, 205-219 (2006).

5 Hino, R., Tomita, M. & Yoshizato, K. The generation of germline transgenic silkworms for the production of biologically active recombinant fusion proteins of fibroin and human basic fibroblast growth factor. *Biomaterials* **27**, 5715-5724 (2006).

6 Ogawa, S., Tomita, M., Shimizu, K. & Yoshizato, K. Generation of a transgenic silkworm that secretes recombinant proteins in the sericin layer of cocoon: production of recombinant human serum albumin. *J Biotechnol* **128**, 531-544 (2007).

7 Zhong, B., Li, J., Chen, J., Ye, J. & Yu, S. Comparison of transformation efficiency of piggyBac transposon among three different silkworm Bombyx

mori Strains. *Acta Biochim Biophys Sin (Shanghai)* **39**, 117-122 (2007).

8 Wen, H. *et al.* Transgenic silkworms (Bombyx mori) produce recombinant spider dragline silk in cocoons. *Mol Biol Rep* **37**, 1815-1821 (2010).

9 Zhao, A. C. *et al.* New and highly efficient expression systems for expressing selectively foreign protein in the silk glands of transgenic silkworm. *Transgenic Res* **19**, 29-44 (2010).

10 Zhuang, L., Wei, H., Lu, C. & Zhong, B. The relationship between internal domain sequences of piggyBac and its transposition efficiency in BmN cells and Bombyx mori. *Acta Biochim Biophys Sin (Shanghai)* **42**, 426-431 (2010).

11 Sato, M. *et al.* Production of scFv-Conjugated Affinity Silk Powder by Transgenic Silkworm Technology. *PLoS One* **7** (2012).

12 Jiang, L. *et al.* Resistance to BmNPV via Overexpression of an Exogenous Gene Controlled by an Inducible Promoter and Enhancer in Transgenic Silkworm, Bombyx mori. *PLoS One* **7** (2012).

13 Jiang, L. *et al.* Identification of a midgut-specific promoter in the silkworm Bombyx mori. *Biochem Bioph Res Co* **433**, 542-546 (2013).

14 Jiang, L. *et al.* Postintegration stability of the silkworm piggyBac transposon. *Insect Biochem Molec* **50**, 18-23 (2014).

15 Duan, J. P. *et al.* Novel female-specific trans-spliced and alternative splice forms of dsx in the silkworm Bombyx mori. *Biochem Bioph Res Co* **431**, 630-635 (2013).

16 Wang, F. *et al.* An optimized sericin-1 expression system for mass-producing

recombinant proteins in the middle silk glands of transgenic silkworms. *Transgenic Res* **22**, 925-938 (2013).

17 Tan, A. J. *et al.* Transgene-based, female-specific lethality system for genetic sexing of the silkworm, Bombyx mori. *P Natl Acad Sci USA* **110**, 6766-6770 (2013).

18 Ma, S. Y. *et al.* Genetic marking of sex using a W chromosome-linked transgene. *Insect Biochem Molec* **43**, 1079-1086 (2013).

19 Deng, D. J. *et al.* The promoter of Bmlp3 gene can direct fat body-specific expression in the transgenic silkworm, Bombyx mori. *Transgenic Res* **22**, 1055-1063 (2013).

20 Tsubota, T. *et al.* Establishment of transgenic silkworms expressing GAL4 specifically in the haemocyte oenocytoid cells. *Insect Molecular Biology* **23**, 165-174 (2014).

21 Xu, H. F. *et al.* Overexpression and functional characterization of an Aspergillus niger phytase in the fat body of transgenic silkworm, Bombyx mori. *Transgenic Res* **23**, 669-677 (2014).

22 Xu, H. F. *et al.* Overexpression of recombinant infectious bursal disease virus (IBDV) capsid protein VP2 in the middle silk gland of transgenic silkworm. *Transgenic Res* **23**, 809-816 (2014).
